# Overexpression of microRNA-95-3p suppresses brain metastasis of lung adenocarcinoma through downregulation of cyclin D1

**DOI:** 10.18632/oncotarget.3886

**Published:** 2015-05-07

**Authors:** Su Jin Hwang, Hye Won Lee, Hye Ree Kim, Hye Jin Song, Dong Heon Lee, Hong Lee, Chang Hoon Shin, Je-Gun Joung, Duk-Hwan Kim, Kyeung Min Joo, Hyeon Ho Kim

**Affiliations:** ^1^ Department of Health Sciences and Technology, Samsung Advanced Institute for Health Sciences and Technology, Sungkyunkwan University, Seoul, Korea; ^2^ Department of Urology, Samsung Medical Center, Sungkyunkwan University School of Medicine, Seoul, Korea; ^3^ Department of Neurosurgery, Institute for Refractory Cancer Research, Samsung Medical Center, Sungkyunkwan University School of Medicine, Seoul, Korea; ^4^ Department of Anatomy and Cell Biology, Sungkyunkwan University School of Medicine, Suwon, Korea; ^5^ Translational Bioinformatics Laboratory, Samsung Genome Institute, Samsung Medical Center, Seoul, Korea; ^6^ Center for Genome Research, Samsung Medical Center, Sungkyunkwan University School of Medicine, Seoul, Korea; ^7^ Samsung Biomedical Research Institute, Institute for Future Medicine, Samsung Medical Center, Seoul, Korea

**Keywords:** microRNA-95-3p, brain metastasis, cyclin D1, lung adenocarcinoma

## Abstract

Despite great efforts to improve survival rates, the prognosis of lung cancer patients is still very poor, mainly due to high invasiveness. We developed brain metastatic PC14PE6/LvBr4 cells through intracardiac injection of lung adenocarcinoma PC14PE6 cells. Western blot and RT-qPCR analyses revealed that PC14PE6/LvBr4 cells had mesenchymal characteristics and higher invasiveness than PC14PE6 cells. We found that cyclin D1 was upregulated, miR-95-3p was inversely downregulated, and pri-miR-95 and its host gene, *ABLIM2*, were consistently decreased in PC14PE6/LvBr4 cells. MiR-95-3p suppressed cyclin D1 expression through direct binding to the 3′ UTR of cyclin D1 mRNA and suppressed invasiveness, proliferation, and clonogenicity of PC14PE6/LvBr4 cells. Ectopic cyclin D1 reversed miR-95-3p-mediated inhibition of invasiveness and clonogenicity, demonstrating cyclin D1 downregulation is involved in function of miR-95-3p. Using bioluminescence imaging, we found that miR-95-3p suppressed orthotopic tumorigenicity and brain metastasis *in vivo* and increased overall survival and brain metastasis-free survival. Consistent with *in vitro* metastatic cells, the levels of miR-95-3p, pri-miR-95, and ABLIM2 mRNA were decreased in brain metastatic tissues compared with lung cancer tissues and higher cyclin D1 expression was involved in poor prognosis. Taken together, our results demonstrate that miR-95- 3p is a potential therapeutic target for brain metastasis of lung adenocarcinoma cells.

## INTRODUCTION

Metastasis is a common feature of malignancy and a leading cause of cancer-related death. Among the many types of cancer, lung cancer is the most common worldwide with the highest mortality rate (less than 20% 5-year survival rate), resulting from high frequency of metastasis [1, 2]. Non-small cell lung cancers (NSCLCs), which account for approximately 85% of all lung cancers, are highly aggressive and easily disseminate to various organs, including the brain. About 40% of patients with lung cancer develop brain metastases during their disease lifetime [3]. Despite progress in treating brain metastases including neurosurgery, radiotherapy, and chemotherapy, the 5-year survival rate of NSCLC remains 15% across all stages of the disease. Moreover, alteration of gene expression and cellular signaling involved in brain metastases remain poorly understood.

MicroRNAs (miRNAs) are a group of highly conserved small non-coding RNAs that participate in post-transcriptional gene regulation via partial complimentary binding to 3′ untranslated regions (UTRs) of target mRNAs. They generally decrease target gene expression through mRNA degradation and translational suppression [4]. Increasing evidence has shown that dysregulation of miRNAs plays critical roles in tumorigenesis and tumor progression including metastasis [5]. For example, the miR-17-92 cluster is highly expressed in lung cancer cells and enhances their proliferation [6]. Conversely, miR-34a is downregulated in NSCLC compared with paired normal lung tissues, and overexpression of miR-34a inhibits NSCLC proliferation [7, 8].

Cyclin D1 is a well-known oncogene that is frequently overexpressed in various cancers including lung cancer [9]. In addition to its functions in cell cycle progression, a recent study demonstrated that cyclin D1 plays a critical role in processes leading to gain of metastatic potential such as migratory and invasive properties. In glioma cells, overexpression of cyclin D1 increased matrix metalloproteinase (MMP) activity and cellular motility, which are responsible for malignant progression of glioma [10]. Many miRNAs regulate expression of cyclin D1 in various cancer cells. Recently, Du et al. reported that miR-545 is downregulated in lung cancer compared with adjacent non-cancerous tissues and inhibits proliferation of lung cancer cells *in vitro* and *in vivo* by targeting cyclin D1 and CDK4 [11].

The goal of this study is to identify novel miRNAs involved in brain metastasis of lung cancer and investigate their roles in controlling metastatic potential. To search for candidate miRNAs, we generated brain metastatic lung cancer cells through left ventricle (LV) injection of lung adenocarcinoma (ADC) cells, PC14PE6. We found that cyclin D1 is upregulated in metastatic cells, and miR-95- 3p, a cyclin D1-targeting miRNA, suppressed invasiveness through downregulation of cyclin D1. Overexpression of miR-95-3p inhibits invasiveness and proliferation of brain metastatic cells *in vitro* and *in vivo*. We also found that the levels of miR-95-3p and its host gene, actin binding LIM protein family member 2 (*ABLIM2*), are lower in brain metastatic lesions than in primary lung cancer, and cyclin D1 expression correlated with poor prognosis. Taken together, our results demonstrate that miR-95-3p is a promising therapeutic target for treatment of brain metastatic lung cancers.

## RESULTS

### Brain metastatic cells, PC14PE6/LvBr4, exhibited mesenchymal characteristics and increased invasive activity

**Figure 1:**
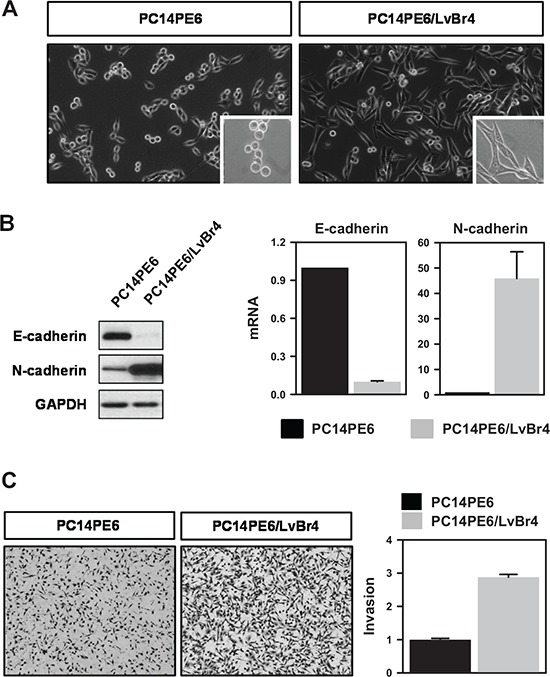
Brain metastatic PC14PE6/LvBr4 cells show mesenchymal features and higher invasive activity than parental PC14PE6 cells **A.** Morphological difference between parental PC14PE6 cells and brain metastatic PC14PE6/LvBr4 cells was assessed by microscopic imaging. **B.** To compare the expression of EMT-related genes, whole-cell lysates and total RNA were prepared from PC14PE6 and PC14PE6/LvBr4 cells. The protein and mRNA levels of epithelial and mesenchymal markers (E-cadherin and N-cadherin, respectively) were determined by western blot (left panel) and RT-qPCR (right panel), respectively. GAPDH mRNA was used for normalization. **C.** The invasive activities of PC14PE6 and PC14PE6/LvBr4 cells were determined by a Transwell invasion assay.

We generated brain metastatic lung cancer cells (PE14PE6/LvBr4) through intracardiac injection of PC14PE6 lung ADC cells. We found that PE14PE6/LvBr4 cells showed morphological features of the epithelial-mesenchymal transition (EMT) such as elongated and fibroblast-like morphology (Figure 1A). During EMT, epithelial cells lose epithelial features such as polarity and cell-cell adhesion and acquire mesenchymal characteristics involved in migration and invasion. We examined expression of epithelial and mesenchymal markers (Figure 1B). PE14PE6/LvBr4 cells showed decreased protein and mRNA expression of E-cadherin and upregulation of N-cadherin, indicating that PE14PE6/LvBr4 cells undergo EMT. Next, we compared the invasive activity of parental and brain metastatic cells using a Transwell invasion assay. PE14PE6/LvBr4 cells exhibited higher invasiveness than parental PC14PE6 cells (Figure 1C). These results revealed that PE14PE6/LvBr4 cells displayed mesenchymal characteristics and significantly increased invasiveness. The median OS duration in mice injected with PC14PE6/LvBr4 cells was significantly shorter than that in mice injected with PC14PE6 cells, likely because of the larger sizes and greater numbers of brain metastases formed by the PC14PE6/LvBr4 cells (data not shown). PC14PE6-LvBr4 cells formed brain metastases in 100% of animals compared to a 40–50% efficiency of the parental lines, demonstrating a significant increase in brain metastatic activity but showed no increase in bone or adrenal gland metastatic activity compared to the parental populations. Authentication of these cell lines was also performed by Short Tandem Repeat (STR) profiling (AmpF/STR Identifier Kit, Applied Biosystems) to confirm that parental and metastatic sublines are genetically identical, thus validating that the metastatic cell line is indeed a derivative of the parental cell line (Supplemental Table 1).

### Cyclin D1 and miR-95-3p were inversely correlated in PE14PE6/LvBr4 cells

**Figure 2:**
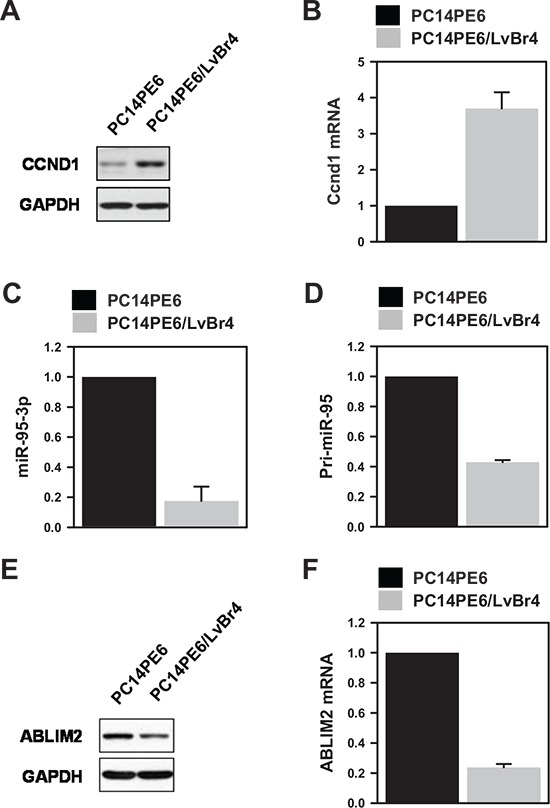
Cyclin D1 is upregulated and miR-95-3p is inversely downregulated in brain metastatic PC14PE6/LvBr4 cells **A–B.** To compare cyclin D1 expression, whole-cell lysates and total RNA were prepared from PC14PE6 and PC14PE6/LvBr4 cells. The protein and mRNA levels of cyclin D1 were determined by western blot (A) and RT-qPCR (B), respectively. **C.** The level of miR-95-3p, a cyclin D1-targeting miRNA, was determined by RT-qPCR using a miRNA-specific looped RT primer and normalized by the level of U6. **D.** The level of primary miR-95 (pri-miR-95) was determined by RT-qPCR. **E–F.** Since miR-95 is located in intron 13 of *ABLIM2*, we compared the levels of ABLIM2 protein (E) and mRNA (F) between parental PC14PE6 and brain metastatic PC14PE6/LvBr4 cells by western blot and RT-qPCR, respectively. GAPDH mRNA was used for normalization.

To investigate the mechanism for gain of metastatic potential, we examined the levels of metastasis-associated genes. We found that cyclin D1 protein and mRNA were highly expressed in PE14PE6/LvBr4 cells relative to PC14PE6 cells (Figure 2A–2B). In contrast to increased cyclin D1, the expression of miR-95-3p was downregulated in PE14PE6/LvBr4 cells (Figure 2C). To check whether the decrease of miR-95-3p resulted from decreased transcription of primary miR-95 (pri- miR- 95), the level of pri-miR-95 was determined by RT-qPCR. Pri- miR-95 expression was also downregulated in PE14PE6/LvBr4 cells (Figure 2D), indicating that the lower level of miR-95-3p arose from decreased transcription of pri-miR-95. Although few miRNAs are regulated by their own transcriptional factors, most miRNAs are transcribed by RNA polymerase II with their host genes. *ABLIM2* is closely associated with cytoskeletal organization and biogenesis and is the host gene of miR-95. Pri-miR-95 is located in intron 13 of *ABLIM2*. Since both the mature and primary forms of miR-95 were downregulated in PE14PE6/LvBr4 cells, we measured expression of *ABLIM2* in PE14PE6/LvBr4 cells. As expected, expression of ABLIM2 protein and mRNA was decreased in PE14PE6/LvBr4 cells compared with PC14PE6 cells (Figure 2E–2F).

### MiR-95-3p inhibited invasive activity by downregulating cyclin D1

**Figure 3:**
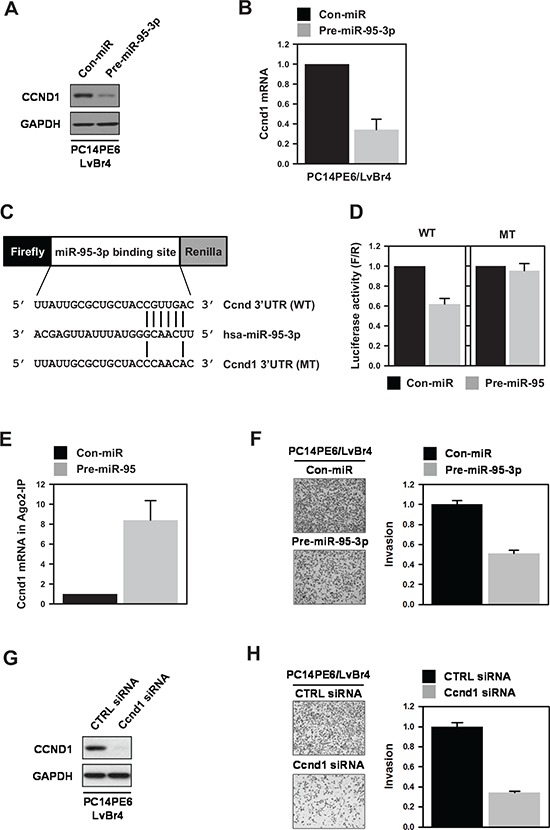
MiR-95-3p suppresses invasiveness of brain metastatic PC14PE6/LvBr4 cells through downregulation of cyclin D1 **A–B.** To investigate the effect of miR-95-3p on cyclin D1 expression, PC14PE6/LvBr4 cells were transfected with control (Con-miR) or pre-miR-95-3p for 48 h and then cyclin D1 protein (A) and mRNA (B) expression were checked by western blot and RT- qPCR, respectively. **C.** Schematic for dual luciferase vector containing a wild-type (WT) or mutated (MT) miR-95-3p-binding sequence was presented. The mutated sequence was constructed by deleting four nucleotides of the seed sequence. **D.** To examine whether miR- 95- 3p directly bound to the 3′ UTR of cyclin D1 mRNA, we constructed a dual luciferase vector as in (C) PC14PE6/LvBr4 cells were transfected with control (Con-miR) or pre-miR-95-3p for 24 h. Cells were resuspended in 12-well plates and then transfected with luciferase vectors. Luciferase activity was determined using a Dual-Glo™ Luciferase Assay System (Promega). Firefly luciferase activity was normalized to that of *Renilla* luciferase. **E.** To verify direct binding of miR-95-3p to the 3′-UTR of cyclin D1 mRNA, we performed Ago2 immunoprecipitation (IP). PC14PE6/LvBr4 cells were transfected as above. The level of cyclin D1 mRNA in IgG or Ago2 IP materials was assessed by RT-qPCR. **F.** The effect of miR-95-3p on invasiveness of PC14PE6/LvBr4 cells was determined using a Transwell invasion assay. **G–H.** To examine whether cyclin D1 is involved in the function of miR-95-3p, PC14PE6/LvBr4 cells were transfected with control (CTRL siRNA) or cyclin D1 siRNA for 48 h. The level of cyclin D1 was determined by western blot (G), and invasive activity was assessed using a Transwell invasion assay (H).

To investigate the effect of miR-95-3p on cyclin D1 expression, PE14PE6/LvBr4 cells were transfected with precursor miR-95-3p (pre-miR-95-3p). The levels of cyclin D1 protein and mRNA decreased in PE14PE6/LvBr4 cells overexpressing miR-95-3p (Figure 3A–3B). To verify that miR-95-3p suppresses cyclin D1 expression, we transfected various lung cancer cells with pre-miR-95- 3p. Overexpression of miR-95-3p resulted in decreased expression of cyclin D1 in all tested cells (Supplemental Figure 1A). The molecular mechanism by which miR-95-3p suppresses cyclin D1 expression was therefore investigated. First, we constructed a luciferase vector (pmirGLO) containing a WT or MT miR-95- 3p binding sequence (Figure 3C). Overexpression of miR- 95- 3p inhibited luciferase expression for the WT sequence but not the MT sequence (Figure 3D). To verify direct interaction between miR-95-3p and the 3′ UTR of cyclin D1 mRNA, we immunoprecipitated Argonaute 2 (Ago2) using cytoplasmic lysates obtained from control or pre-miR-95-3p-transfected PC14PE6/LvBr4 cells (Figure 3E). We found that cyclin D1 mRNA was enriched in Ago2 IP materials, and overexpression of miR-95-3p increased the enrichment of cyclin D1 mRNA in Ago2 IP relative to control miRNA-transfected cells. These results reveal that miR-95-3p suppresses cyclin D1 expression through direct interaction with the 3′ UTR of cyclin D1 mRNA.

To address the functional role of miR-95-3p in metastatic potential, we examined whether miR-95-3p affects invasiveness of PC4PE6/LvBr4 cells. Invasive activity was diminished by overexpression of miR-95-3p in PC14PE6/LvBr4 cells (Figure 3F). Similarly, miR-95-3p inhibited invasiveness of H1299 lung cancer cells (Supplemental Figure 1B). To investigate whether suppression of cyclin D1 was involved in the function of miR-95-3p, we assessed invasive activity in cyclin D1-silenced metastatic cells. To silence cyclin D1, we used two siRNAs that target different cyclin D1 sequences. Both siRNAs inhibited the invasiveness of PC14PE6/LvBr4 cells (Figure 3G–3H and Supplemental Figure 2). Since cyclin D1 siRNA #2 showed more inhibitory effect on invasive activity, we represented results using cyclin D1 siRNA #2. These results indicate that increased expression of cyclin D1 is involved in gain of metastatic potential and, furthermore, that miR-95-3p inhibited invasiveness by suppressing cyclin D1 expression.

### MiR-95-3p inhibits clonogenicity and proliferation of metastatic cells *in vitro* and *in vivo*

**Figure 4:**
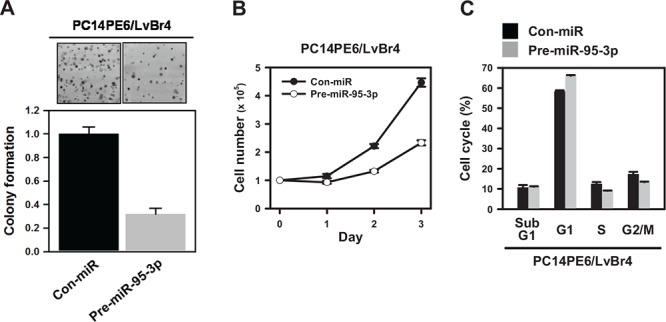
MiR-95-3p inhibits proliferation of brain metastatic PC14PE6/LvBr4 cells **A.** To examine the effect of miR-95-3p on clonogenicity, a colony-forming assay was performed. Briefly, cells transfected with control (Con-miR) or pre-miR-95-3p were seeded into 6-well plates and then cultured for two weeks. Clonogenicity was determined by counting stained colonies. **B.** Equal numbers of transfected cells were seeded into 12-well plates, and the number of cells was counted every 24 h. **C.** Cells transfected with control (Con-miR) or pre-miR-95-3p were fixed and stained with propidium iodide (PI). Cell cycle analyses were performed using FACS, and the population of each cell cycle was calculated using FACS verse.

The effect of miR-95-3p on clonogenicity was evaluated using a colony-forming assay. PC14PE6/LvBr4 cells transfected with pre-miR-95-3p showed fewer and smaller colonies than Con-miR-transfected cells, demonstrating that miR-95-3p diminishes clonogenicity in PC14PE6/LvBr4 cells (Figure 4A). To investigate the effect of miR-95-3p on proliferation, PC14PE6/LvBr4 cells were transfected as described above. Equal numbers of transfected cells were cultured in 6-well plates, and viable cells were counted every 24 h. Overexpression of miR-95-3p inhibited the proliferation of PC14PE6/LvBr4 cells (Figure 4B). Moreover, flow cytometry analysis revealed that the proportion cells in the G1 phase was slightly increased in PC14PE6/LvBr4 cells overexpressing miR-95-3p, suggesting that miR-95-3p diminished proliferation partially through weak G1 arrest (Figure 4C).

### Ectopic expression of cyclin D1 restores miR-95-3p-mediated inhibition of invasiveness and clonogenicity

**Figure 5:**
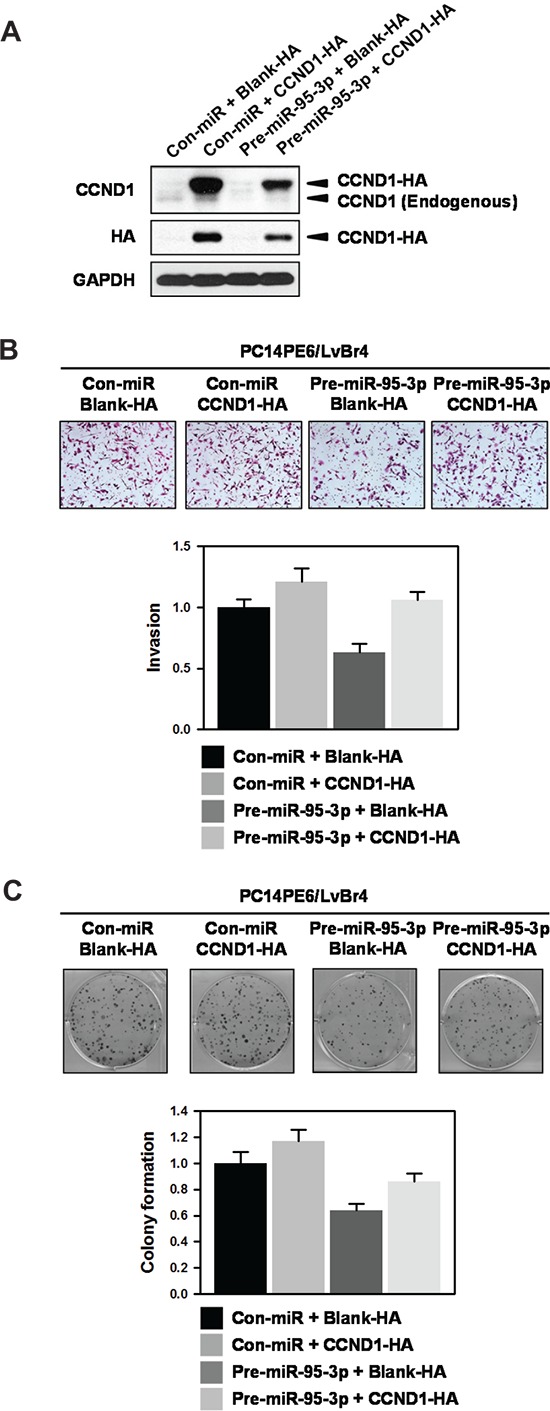
Ectopic expression of cyclin D1 restores miR-95-3p-mediated inhibition of invasiveness and colony formation **A.** To investigate the effect of ectopic expression of cyclin D1 on miR-95-3p-mediated inhibition of invasiveness and colony formation, PC14PE6/LvBr4 cells were transfected with pre-miR-95-3p, CCND1-HA vector, or both for 48 h. Whole-cell lysates were prepared and the levels of endogenous and ectopic cyclin D1 were determined by Western blot analysis using cyclin D1- and HA-specific antibody, respectively. GAPDH was used as loading control. **B.** Equal numbers of transfected cells as described above were inoculated into a Transwell to examine invasiveness. Invaded cells were stained and counted under microscope. **C.** Equal numbers of transfected cells were seeded into 6-well plates and then cultured for two weeks. Clonogenicity was determined by counting stained colonies.

From above results, we found that miR-95-3p inhibits invasiveness, proliferation, and clonogenicity by suppressing cyclin D1 expression. To verify that decreased expression of cyclin D1 is involved in the effects of miR-95-3p, we investigate whether ectopic expression of cyclin D1 restores miR-95-3p-mediated inhibition of invasiveness and clonogenicity. Briefly, PC14PE6/LvBr4 cells were transfected with pre-miR-95-3p, CCND1-HA vector, or both for 48 h and the levels of endogenous and ectopic cyclin D1 were examined by Western blot analysis (Figure 5A). Invasiveness (Figure 5B) and clonogenicity (Figure 5C) of PC14PE6/LvBr4 cells was suppressed by overexpression of miR-95-3p as shown earlier and ectopic expression of cyclin D1 restored miR-95-3p-mediated inhibition of invasiveness and colony formation, demonstrating that suppression of cyclin D1 expression is responsible for the function of miR-95-3p.

### Implications of miR-95-3p in the tumorigenesis and metastasis of lung ADC *in vivo*

**Figure 6:**
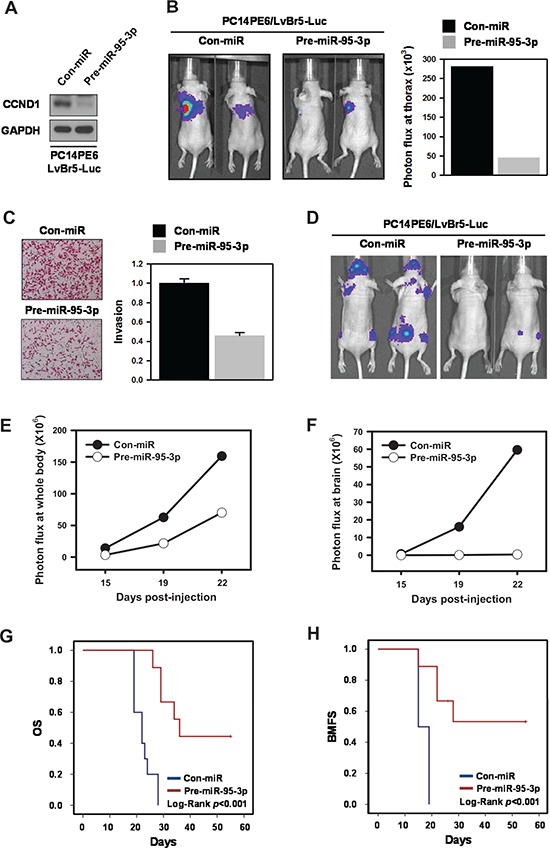
MiR-95-3p inhibits tumorigenesis and brain metastasis, thereby promoting overall survival (OS) and brain metastasis-free survival (BMFS) For bioluminescence imaging, luciferase-tagged brain metastatic cells (PC14PE6/LvBr5-Luc) were generated. **A.** To measure the suppression of cyclin D1 by miR-95-3p, PC14PE6/LvBr5-Luc cells were transfected with control (Con-miR) or pre-miR-95-3p for 48 h, and the level of cyclin D1 was determined by western blot. **B.** To examine the effect of miR-95-3p on proliferation *in vivo*, transfected PC14PE6/LvBr5-Luc cells were injected orthotopically into the lungs of mice. Bioluminescence images were acquired with the IVIS Spectrum imaging system (PerkinElmer) and quantified by measurement of photon flux (photons/s/cm^2^/steradian) using the Living Image Software package (Perkin Elmer/Caliper Life Sciences). **C.** PC14PE6/LvBr5-Luc cells were transfected with control (Con-miR) or pre-miR-95-3p for 48 h, and equal numbers of cells were inoculated into a Transwell to examine invasiveness. Invaded cells were stained and counted under microscope. **D.** To investigate the effect of miR-95-3p on brain metastasis *in vivo*, transfected PC14PE6/LvBr5-Luc cells were directly injected into the left ventricle of the heart. Bioluminescence images were acquired and quantified. Incidence of whole body **E.** and brain metastasis **F.** was quantified based on the luminescent signal at a given time point. **G–H.** Kaplan-Meier curves and *p*-values for overall survival (OS) and brain metastasis-free survival (BMFS) were analyzed in systemic metastasis models.

To investigate whether miR-95-3p affects primary lung ADC tumorigenesis *in vivo*, we generated PC14PE6/LvBr5-Luc cells for bioluminescence imaging. First, we examined whether miR-95-3p also suppressed cyclin D1 expression in PC14PE6/LvBr5-Luc cells and found that cyclin D1 was downregulated by miR-95-3p in PC14PE6/LvBr5-Luc cells (Figure 6A). Additionally, miR-95-3p decreased proliferation rate and suppressed clonogenicity of PC14PE6/LvBr5-Luc cells without inducing apoptotic cell death (Supplemental Figure 3). Next, we measured the effect of miR-95-3p on orthotopic tumorigenicity *in vivo*. PC14PE6/LvBr5-Luc cells (2 × 10^6^) transfected with Con-miR (*N* = 4) or pre-miR-95-3p (*N* = 6), were implanted into the lungs of nude mice. Eleven days after cancer cell inoculation, the lung colonization signal was reduced in cells expressing miR-95-3p compared to their counterparts, indicating that miR-95-3p expression inhibits primary lung tumor growth (Figure 6B, *p* = 0.23). This result was not statistically significant due to the small number of mice. These results are consistent with those of the *in vitro* cell proliferation assay, which firmly validates a significant role of miR-95-3b in lung tumorigenesis.

Our results strongly suggest that miR-95-3p has an anti-proliferative effect *in vitro* and *in vivo*. As expected from the decreased expression of cyclin D1, overexpression of miR-95-3p inhibited invasiveness of PC14PE6/LvBr5-Luc cells (Figure 6C). Therefore, we examined the effect of miR-95-3p on brain metastasis of PC14PE6/LvBr5-Luc by intracardiac injection (2 × 10^5^ PC14PE6-LvBr5-Luc/Con-miR, *N* = 12 or PC14PE6-LvBr5-Luc/pre-miR-95-3p, *N* = 9). MiR-95- 3p significantly reduced metastasis of PC14PE6/LvBr5-Luc cells to the whole body and brain, as reflected by bioluminescence signals 19 and 22 days post-injection (Figure 6D–F). We also found that miR-95-3p prolonged OS and BMFS (Figure 6G–H, respectively, all Log rank *p* < 0.001). These *in vivo* results indicated that miR-95- 3p could suppress metastasis of lung ADC cells, which is mediated by decreased cell proliferation, invasion, and clonogenicity.

### MiR-95-3p and *ABLIM2* were downregulated in brain metastatic lesions with lung cancer, and cyclin D1 expression correlated with poor prognosis

**Figure 7:**
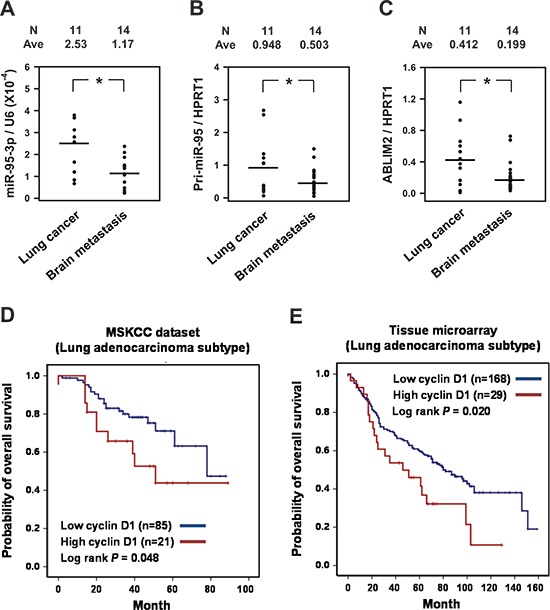
Decreased expression of miR-95-3p and *ABLIM2* in brain metastatic tissue and correlation of cyclin D1 with the survival of patients with lung adenocarcinoma (ADC) **A–C.** Surgical specimens and clinical records were obtained from 25 lung ADC patients who underwent surgical removal of primary tumors (*N* = 11) or brain metastases (*N* = 14). The levels of miR- 95-3p, pri-miR-95, and *ABLIM2* were determined by RT-qPCR. *p < 0.05. **D.** Kaplan-Meier analysis for overall survival (OS) of 107 lung ADC patients obtained from Memorial Sloan-Kettering Cancer Center (MSKCC set 1; Affymetrix HG-U133A). The patients were divided into *high* and *low* cyclin D1 groups based on mRNA expression levels. **E.** Kaplan-Meier analysis for overall survival (OS) of 197 patients with pathologically proven lung ADC at the Samsung Medical Center. Only nuclear staining of cyclin D1 was considered to define positive staining, and the immunoreactivity of cyclin D1 was categorized as low (weak to moderate) or high (strong). Overall survival was calculated from the date of histological diagnosis to death.

To further support our findings, we determined the levels of miR-95-3p, pri-miR-95, and *ABLIM2* in surgical specimens obtained from 25 patients with lung ADC who underwent surgical removal of primary tumors (*N* = 11) or brain metastases (*N* = 14). Similar to the *in vitro* findings, the levels of miR-95-3p and pri-miR-95 were downregulated in brain metastatic lung cancer tissues (Figure 7A–B, respectively). Furthermore, *ABLIM2* was also decreased in brain metastatic lesions relative to primary lung cancer tissues (Figure 7C).

To determine the prognostic significance of cyclin D1 expression in patients with lung ADC, we determined cyclin D1 mRNA and protein expression levels in the MSKCC public dataset (*N* = 106) and by TMA, respectively. Notably, high transcriptional expression of cyclin D1 (*N* = 21) was associated with significantly poor OS compared to the group that expressed a low level of cyclin D1 mRNA (*N* = 95) in the MSKCC lung ADC set (Figure 7D, log-rank test, *p* = 0.048). Consistent with this finding, measurement of cyclin D1 expression in 197 lung ADCs by IHC showed that cyclin D1-high patients had significantly worse clinical prognosis than cyclin D1-low patients (Figure 7E, log-rank test, *p* = 0.020). These findings reveal a significant association between the overexpression of cyclin D1 regulated by miR-95-3p in primary lung ADC tumors and OS.

## DISCUSSION

Metastatic cancers are difficult to cure or incurable and account for more than 90% of cancer-related deaths [12]. To investigate the roles of miRNA in gain of metastatic potential, we obtained metastatic cells through LV injection of PC14PE6 lung ADC cells. We found that cyclin D1 was upregulated in metastatic cells with higher invasive activity compared to parental cells. Conversely, cyclin D1-targeting miR-95-3p was decreased in metastatic cells. We also found that miR- 95-3p suppressed the expression of cyclin D1 by direct binding to the 3′ UTR of its mRNA.

Cyclin D1, a member of the cyclin family, plays a critical role in control of G1 progression. It is synthesized in the cytoplasm and assembled with its partners such as cyclin-dependent kinase (CDK) 4 and CDK6 in the nucleus to drive cells from G1 to S phase. In addition to regulating the cell cycle, Cyclin D1 is a well-known oncogene that is frequently overexpressed in various cancer cells. Recent evidence indicates that deregulation of cyclin D1 is involved in cancer progression including metastasis [13]. Moreover, cyclin D1 is reported to regulate many genes governing cellular adhesion and migration independently of CDKs. Li et al. reported that introduction of wild-type cyclin D1 into cyclin D1^+/−^ mouse embryonic fibroblasts (MEFs) suppresses the expression of thrombospondin 1 (TSP1) and Rho-activated kinase II (ROCKII), which increases the migratory activity of MEFs [14]. Therefore, cyclin D1 increases cellular migration through downregulation of TSP1 and ROCKII. Furthermore, cyclin D1 enhances the expression and activity of MMPs, thus increasing invasiveness [10]. Recently, interaction between cyclin D1 and a cell cycle inhibitor, p27, was shown to be involved in cyclin D1- mediated migration of MEFs. Enhanced migration by cyclin D1 is abrogated by mutation of K112 or deletion of the p27-binding site, which are crucial for interaction with p27 [15]. In addition to p27, cyclin D1 must interact with p21 for transforming growth factor (TGF)-β-mediated migration and invasion. Cyclin D1 is specifically induced by TGF-β and is responsible for TGF-β-mediated migration and invasion in cooperation with p21 [16].

Cyclin D1 has two functional roles in pathogenesis of NSCLC [9]. The cyclin D1 locus is amplified in more than 30% of cancers, and the cyclin D1 protein is frequently overexpressed in both invasive cancers and pre-invasive lesions of the bronchial epithelia. Moreover, increasing evidence supports a role for cyclin D1 and CDK4 in the promotion of cancer cell proliferation, and they are more abundant in lung tumor tissue than normal lung tissue [11]. Several oncogenes such as HER2 and Ras activate the cyclin D1 promoter, and silencing of cyclin D1 inhibits their oncogenic signaling [17]. In addition to genomic regulation, transcriptional gene regulation by miRNAs is a main mechanism for dysregulation of metastasis-associated genes. Several miRNAs can inhibit cyclin D1 expression, and most studies have investigated the anti-proliferative effects of miRNAs. Analysis of the expression of 450 miRNAs in lung tumor tissues and adjacent normal tissues indicated that miR-545 is frequently downregulated in lung tumor tissues and suppresses proliferation of lung cancer cells [11]. MiR- 9 was reported to inhibit the proliferation, invasion, and metastasis of gastric cancer cells through downregulation of cyclin D1 [18].

Paradoxically, miR-95 was reported to promote cell proliferation in colorectal carcinoma and NSCLC, indicating that miR-95 is an oncogenic miRNA [19, 20]. Based on immunoprecipitation (IP) experiments, 43 genes are validated as targets of miR-95-3p (provided through DIANA TOOLS). However, only two of them are reported: sorting nexin 1 (SNX1) and sphingolipid phosphatase (SGPP1). Mechanistic investigation showed that miR-95 suppresses expression of SNX1 by directly targeting its 3′ UTR in colorectal and lung cancer cells. Furthermore, miR-95 induces chemo- and radioresistance in NSCLC [20]. In contrast to its proliferative effects, miR-95 is reported to play a critical role in the anticancer activity of Brucein D, a compound isolated from *Brucea javanica* fruit [21]. The level of miR-95 is upregulated by treatment with Brucein D in hepatocellular carcinoma and suppresses expression of CUG triplet repeat RNA-binding protein 2 (CUGBP2), which is responsible for the anticancer effect of Brucein D. In our results, miR-95-3p inhibits proliferation of brain metastatic PC14PE6/LvBr4 cells. This effect may arise from genetic alterations to growth-associated genes and miRNAs during metastasis that may lead to different cellular responses to miRNAs. MiRNAs are also known to provide new therapeutic targets for many diseases including cancers. Based on genome-wide profile of differentially expressed miRNAs, applications of miRNA have expanded to cancer diagnosis. In colorectal cancer (CRC) patient, miR-95 was reported to be overexpressed in preoperative serum sample of CRC patients [22], suggesting that miR-95 has a potential for cancer diagnosis.

More than half of total miRNAs are located in the introns of coding or non-coding transcripts. Although the regulatory mechanism of biogenesis is slightly different between exonic and intronic miRNAs, most miRNAs are transcribed in parallel with their host genes [23]. Our results demonstrate that miR-95, which is located in intron 13 of *ABLIM2*, is downregulated in PC14PE6/LvBr4 cells and brain metastatic tissues. The level of the *ABLIM2* transcript was consistently decreased in brain metastasis *in vitro* and *in vivo*. To metastasize from the primary site to an adjacent site, cancer cells reorganize their cytoskeletal structures. Since actin-binding proteins play important roles in actin polymerization, many studies have focused on their functions in regulating metastatic potential. During metastatic processes, the level of actin-binding proteins is dynamically controlled. Expression of Arp2/3 is reduced during gastric cancer progression [24]. Conversely, we previously reported that α-smooth muscle actin (ACTA2) is responsible for the metastatic potential of lung ADC cells [25]. Our results demonstrate that decreased expression of *ABLIM2* results in downregulation of miR-95-3p in brain metastatic PC14PE6/LvBr4 cells, which suggests that alterations in the levels of actin-binding protein may affect the expression of metastasis-associated genes including cyclin D1 through regulation of miRNAs. Taken together, our results demonstrate that miR-95-3p is a promising therapeutic target for brain metastases of lung ADC.

## MATERIALS AND METHODS

### Generation of brain metastatic sublines PC14PE6/LvBr4 and PC14PE6/LvBr5-Luc

The brain is the most common site of metastasis from NSCLCs [26]. We carried out *in vivo* selection experiments to isolate metastatic cells from the parental PC14PE6 human lung ADC cell lines, the cells that most actively seed a tumor mass from circulation. PC14PE6 cells were established from a pleural effusion that developed in a nude mouse injected intravenously with parental human lung ADC PC14 cells [27]. To identify the subpopulation of parental PC14PE6 cells capable of forming experimental brain metastasis in nude mice, we initially injected mice with PC14PE6 cells via the LV. The body weights of the animals were measured daily, and the mice were euthanized immediately after 25% weight loss or demonstration of gait and balance difficulties. Metastatic cells were isolated and expanded from harvested brains as previously described [28], then injected into the LV of nude mice. We repeated this injection-isolation-expansion cycling three additional times and designated the final cell line established using this process as PC14PE6-LvBr4, yielding PC14PE6 tumor cells that were recycled to the brain.

To generate PC14PE6-LvBr5-Luc stable cells, pre-made Lentiviral Expression Particles for firefly luciferase (LVP325, Amsbio) were transduced immediately into PC14PE6/LvBr4 cells. For selection, transduced cells were cultured for two weeks in media containing puromycin (3 μg/mL), and stable clones were assayed for luciferase activity using the Bright-GloTM luciferase assay (Promega). These PC14PE6/LvBr4-Luc cells were then subjected to one round of *in vivo* selection via intracardiac injection, yielding PC14PE6/LvBr5-Luc cell populations with greater brain metastatic activity and validated *in vivo* luciferase activity.

### Cell culture and transfection

PC14PE6 and its derivative cells, PC14PE6/LvBr4 and LvBr5-Luc, were maintained at 37°C and 5% CO_2_ in DMEM (Hyclone) supplemented with 10% fetal bovine serum (FBS) and 1% antibiotic-antimycotic solution (GIBCO-BRL) [25].

For transfection, cells were plated at a density of 5 × 10^5^ cells/dish and transfected with the indicated small interfering RNAs (siRNAs) or pre-miRNAs with appropriate control siRNA or miRNA, respectively, using Lipofectamine2000 (Invitrogen) according to the manufacturer's protocol. Control and cyclin D1-targeting siRNAs were synthesized by ST Pharm (South Korea), and pre-miR-96-3p was purchased from Ambion and used for overexpression of miR-95-3p. To perform rescue experiments, Rc/CMV cyclin D1 HA, a gift from Philip Hinds (Addgene plasmid #8948), was used for overexpression of cyclin D1 [29].

### Western blot and real-time quantitative polymerase chain reaction (RT-qPCR) analyses

For western blot analyses, RIPA buffer containing protease inhibitors and phosphatase inhibitors (Roche) was used to prepare whole-cell lysates [30]. Briefly, equal amounts of lysate were separated by SDS-polyacrylamide gel electrophoresis (SDS-PAGE) and then transferred to PVDF membranes (Millipore). After blocking with 5% bovine serum albumin (BSA), membranes were incubated with the indicated primary antibodies overnight, then with the appropriate secondary antibodies. Antibodies for cyclin D1 (Cell Signaling Technology), ABLIM2, GAPDH (Abcam), E-cadherin, N-cadherin (BD Bioscience), and ZEB1 (Bethyl Laboratories) were used in this study.

Total RNA was isolated using TRIzol reagent (Invitrogen) according to the manufacturer's instructions and used as template to synthesize cDNA. The mRNA level was quantified by RT-qPCR (ABI Prism 7600) using power SYBR^®^ Green PCR Master Mix (Applied Biosystems). The following primers were used for amplification: Cyclin D1, forward (5′-CCGTCCATGCGGAAGATC-3′) and reverse (5′-ATGGCCAGCGGGAAGAC-3′); ABLIM2, forward (5′-AGCTGACTATCACGCCAAGT-3′) and reverse (5′-GGAAGGGTGGTAGTGCTTCT-3′); primary miR-95, forward (5′-CTGGTGGAGGGATGGATGAA-3′) and reverse (5′-GGCCCGATCACAAACTCATC-3′); HPRT1, forward (5′-GACACTGGCAAAACAATGCA-3′) and reverse (5′-CTTCGTGGGGTCCTTTTCACC-3′); and GAPDH, forward (5′-TGCACCACCAACTGCTTAGC-3′) and reverse (5′-GGCATGGACTGTGGTCATGAG-3′). GAPDH mRNA was used for normalization.

### Luciferase assay

For the cyclin D1 3′ UTR luciferase assay, we constructed dual luciferase vectors (pmirGLO dual-luciferase vector) containing the wild-type (WT) or mutated (MT) miR-95-3p binding site in the cyclin D1 3′ UTR. Mutated binding sites were constructed through substitution of four nucleotides in the seed region. PC14PE6/LvBr4 cells were transfected with control (Con-miR) or pre-miR-95-3p for 24 h, resuspended in 6-well plates, and transfected with luciferase vectors (MT and WT) for 24 h. Luciferase activity was determined using a Dual-Glo™ Luciferase Assay System (Promega). The *Firefly* luciferase activity was normalized to that of *Renilla* luciferase.

### Determination of invasive activity

Invasive activity was determined using Matrigel Transwells (BD Biocoat Matrigel Invasion Chamber). Briefly, equal numbers of cells in serum-free media were added to the upper chamber. Cellular invasion was triggered by addition of complete media containing 10% FBS to the lower chamber. After 24 h, cells were fixed with 100% methanol and stained with 0.1% hematoxylin and eosin (H&E). Quantification of invasiveness was performed by counting the number of invaded cells from more than ten fields [30].

### Assay for clonogenicity, proliferation, and cell cycle

To determine clonogenicity, a colony-forming assay was performed. Briefly, ~300 cells were seeded into each well of 6-well plates and cultured in complete medium for two weeks. After cells were stained with 0.2% crystal violet, the number of stained colonies was counted. In addition to clonogenicity, we examined cell proliferation. Transfected cells were seeded into 6-well plates at a density of 1 × 10^5^ cells/well. Cell proliferation was assessed by counting viable cells every 24 h. For cell cycle analyses, transfected cells were fixed with 70% ice-cold ethanol and then stained with propidium iodide (PI) solution containing RNase A. The stained cells were quantified using a fluorescence-activated cell sorter (FACS, FACS verse, BD) and the population of each cell cycle was analyzed by BD FACS suite software.

### Tumorigenicity and metastasis assay *in vivo* using bioluminescence imaging

All animals received humane care in compliance with the “Guide for the Care and Use of Laboratory Animals” prepared by the Institute of Laboratory Animal Resources published by the National Institutes of Health and according to the Animal Experiment Guidelines of Samsung Biomedical Research Institute. The effect of miR-95-3p on the tumorigenic and metastatic potential of lung ADC cells was analyzed in orthotopic and systemic metastasis *in vivo* models via intra-lung and intracardiac injection, respectively. For the orthotopic model, 6–8 week-old female BALB/c nude mice were anesthetized in the right lateral decubitus position. After making a 3 mm incision at the lateral dorsal axillary line just below the inferior border of the scapula, we injected transfected cancer cells (2 × 10^6^ cells in 40 μL Hank's Balanced Slat Solution (HBSS)) into the left lung. For the experimental metastasis *in vivo* model, transfected cancer cells (2 × 10^5^ in 100 μL of HBSS) were directly injected into the LV of the heart. The body weights of the animals were measured daily, and an autopsy was performed immediately after 25% weight loss.

Bioluminescence images were collected to assess the growth and metastasis of implanted tumor cells. To quantify the *in vivo* bioluminescence signal, mice were anesthetized with isoflurane before *in vivo* imaging, and D-luciferin solution (*in vivo* imaging solutions, PerkinElmer, 150 mg/kg in PBS) was injected intravenously for both orthotopic and systemic xenografts. Bioluminescence images were acquired with the IVIS Spectrum imaging system (PerkinElmer) 2–5 min after injection, and the captured images were quantified using the Living Image Software package (Perkin Elmer/Caliper Life Sciences) by measuring the photon flux (photons/s/cm^2^/steradian) within a region of interest (ROI) drawn around the bioluminescence signal. Incidence of metastasis was quantified based on the luminescent signal at a given time point and presented as Kaplan-Meir curves. Kaplan-Meier curves and *p*-values for overall survival (OS) and brain metastasis-free survival (BMFS) were analyzed in systemic metastasis models.

### Lung ADC surgical samples

Surgical specimens and clinical records were obtained from 25 lung ADC patients who underwent surgical removal of primary tumors (*N* = 11) or brain metastases (*N* = 14) at Samsung Medical Center from 2008–2012. Written informed consent was obtained from all participants, and tissue collection was approved by the Samsung Medical Center Institutional Review Board (IRB) (No. 2010-04-004).

### Lung ADC gene expression datasets

A publicly available data set containing a cohort of 107 lung ADCs from the Memorial Sloan-Kettering Cancer Center (MSKCC set 1; Affymetrix HG-U133A) was down-loaded from http://cbio.mskcc.org/Public/lung array data [31]. In order to remove systematic biases, the expression measurements were converted to z-scores for all genes prior to merging. The patients were divided into high (upper 25^th^ percentile; first quartile) and low (remaining 26^th^ – 100^th^ percentile) cyclin D1 group on the basis of levels of mRNA expression, and the Kaplan-Meier survival function was plotted.

### Immunohistochemistry (IHC) on lung ADC tissue microarray (TMA)

The surgically removed samples were collected from 197 patients with pathologically proven lung ADC at the Samsung Medical Center between November 1994 and August 2004 after obtaining appropriate IRB permission and after written informed consent was obtained from all patients. Seventy-seven of 197 patients had stage I lung ADC (39.1%), 78 patients had stage II lung ADC (39.6%), 37 patients had stage III lung ADC (37%), and 4 patients had stage IV lung ADC (2.5%). OS was calculated from the date of histological diagnosis to death, and the median survival time was 66 months (interquartile range 25–91 months). TMA sections generated from the surgical samples were immunohistochemically stained as previously described with rabbit monoclonal antibody against cyclin D1 (Clone Sp4, Thermo Scientific Lab Vision) [32]. Only nuclear staining was considered to define positive staining, and the immunoreactivity of cyclin D1 was categorized as low (weak to moderate) or high (strong).

## SUPPLEMENTARY FIGURES AND TABLE


