# Supplementary material for: Overexpression of microRNA-95-3p suppresses brain metastasis of lung adenocarcinoma through downregulation of cyclin D1
Source: Oncotarget. 2015 May 7;6(24):20434–48. doi: 10.18632/oncotarget.3886 (PMC4653016; doi:10.18632/oncotarget.3886)
Supplement: Supplementary file 1 [file oncotarget-06-20434-s001.pdf]

SUPPLEMENTARY FIGURES AND TABLE

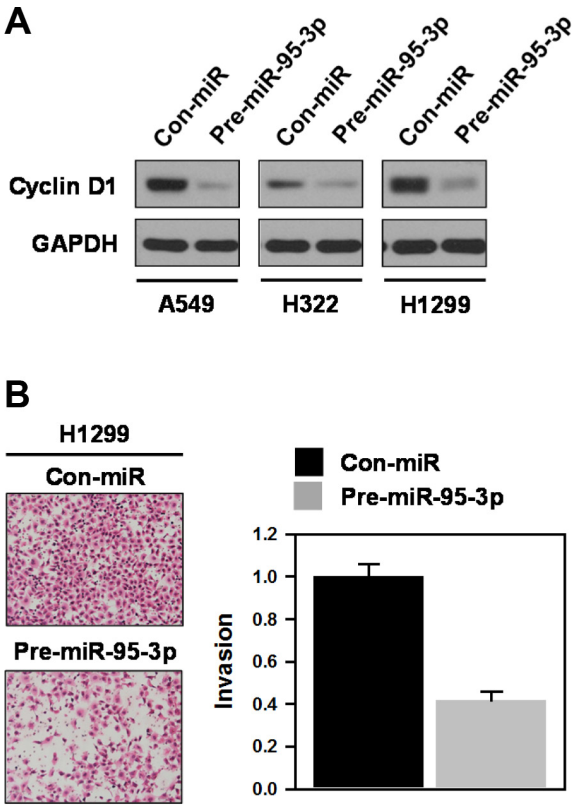

Supplementary Figure S1: Overexpression of miR-95-3p suppressed cyclin D1 expression and inhibited invasiveness in various lung cancer cells. A. To verify the effect of miR-95-3p on cyclin D1 expression, A549, H322, and H1299 cells were transfected with control (Con-miR) or pre-miR-95-3p for 48 h and then whole-cell lysates were prepared using RIPA buffer. Equal amount of protein was separated by SDS-PAGE and the level of cyclin D1 and GAPDH was determined by Western blot. **B.** H1299 cells were transfected as described above and invasive activity was determined by Transwell invasion assay.

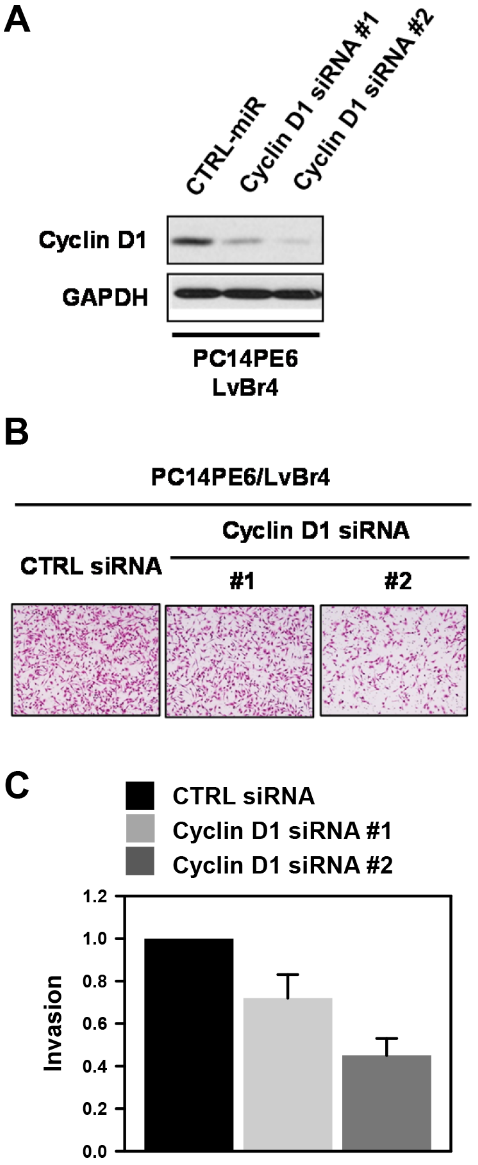

Supplementary Figure S2: Silencing of cyclin D1 inhibits invasive activity of brain metastatic PC14PE6/LvBr4 cells. **A.** To investigate the effect of cyclin D1 silencing on invasiveness, PC14PE6/LvBr4 cells were transfected with control (CTRL siRNA) or two different siRNA for cyclin D1 (#1 and #2) for 48 h and the level of cyclin D1 and GAPDH was determined by Western blot. **B-C.** Invasiveness of transfected cells was assessed using Transwell invasion assay. Invaded cells were stained with H&E and counted under microscope.

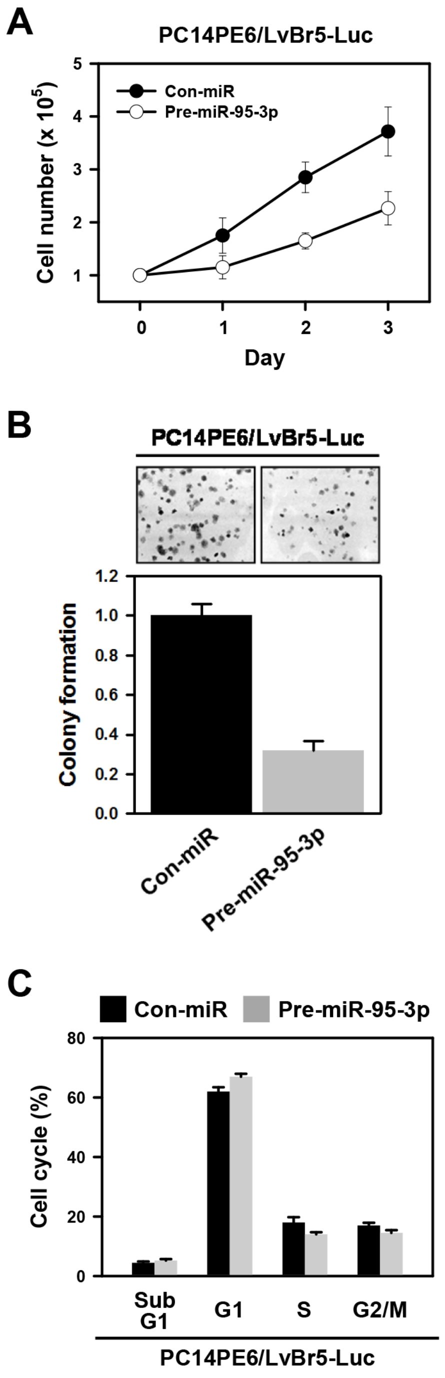

Supplementary Figure S3: Overexpression of miR-95-3p inhibited proliferation and suppressed colony formation of PC14PE6/LvBr5-Luc cells without inducing apoptotic cell death. A. To investigate the effect of miR-95-3p on proliferation of PC14PE6/LvBr5-Luc cells, cells were transfected with control (Con-miR) or pre-miR-95-3p for 48 h. Equal numbers of transfected cells were seeded into 12-well plates, and the number of cells was counted every 24 h. **B.** Transfected cells with control (Con-miR) or pre-miR-95-3p were seeded into 6-well plates and then cultured for two weeks. Clonogenicity was determined by counting stained colonies. **C.** Cells transfected with control (Con-miR) or pre-miR-95-3p were fixed and stained with propidium iodide (PI). Cell cycle analyses were performed using FACS, and the population of each cell cycle was calculated using FACS verse.

Supplementary Table S1. Authentication of parental PC14PE6 and brain metastatic PC14PE6/LVBr4 cells by Short Tandem Repeat (STR) profiling

Locus	Chromosome Location	PC14PE6	PC14PE6/LvBr4
D8S1179	8	13	13
D21S11	21q11.2-q21	33.2	33.2
D7S820	7q11.21-22	9	9
CSF1PO	5q33.3-34	13	13
D3S1358	3p	15	15
TH01	11p15.5	9	9
D16S539	16q24-qter	12	12
D2S1338	2q35-37.1	17, 19	17,19
D19S433	19q12-13.1	13	13
vWA	12p12-pter	16, 18, 19	16, 18, 19
TPOX	2p23-2per	8	8
D18S51	18q21.3	14	14
D5S818	5q21-31	13, 14	13, 14
FGA	4q28	24	24
